# Positive selection in coding regions and motif duplication in regulatory regions of bottlenose dolphin MHC class II genes

**DOI:** 10.1371/journal.pone.0203450

**Published:** 2018-09-25

**Authors:** Heidi J. T. Pagán, Tatiana Ferrer, Greg O’Corry-Crowe

**Affiliations:** Harbor Branch Oceanographic Institute, Florida Atlantic University, Fort Pierce, FL, United States of America; Universidad de los Andes, COLOMBIA

## Abstract

The vertebrate immune response is mediated through highly adaptive, quickly evolving cell surface receptors, the major histocompatibility complex (MHC). MHC molecules bind and present a diverse array of pathogenic molecules and trigger a cascade of defenses. Use of MHC variation as a marker for population health has also evolved quickly following advances in sequencing methods. We applied a combination of traditional and next generation sequencing methodology to characterize coding (peptide binding region) and regulatory (proximal promoter) sequence variation in MHC Class II *DQA* and *DQB* genes between estuarine and coastal populations of the bottlenose dolphin, *Tursiops truncatus*, an apex predator whose health status is indicative of anthropogenic impacts on the ecosystem. The coding regions had 10 alleles each at *DQA* and *DQB*; the promoters had 6 and 7 alleles at *DQA* and *DQB*, respectively with variation within key regulatory motifs. Positive selection was observed for the coding regions of both genes while both coding and promoter regions exhibited geographic differences in allele composition that likely indicates diversifying selection across habitats. Most notable was the discovery of a complete duplication of a 14-bp T-box motif in the *DQA* promoter. Four class II promoter regions (*DQA*, *DQB*, *DRA*, *DRB*) were characterized in species from four cetacean families (Delphinidae, Monodontidae, Lipotidae, and Physeteridae) and revealed substantial promoter structural diversity across this order. Peptide binding regions may not be the only source of adaptive potential within cetacean MHC for responding to pathogenic threats. These findings are the first analysis of cetacean MHC regulatory motifs, which may divulge unique immunogenetic strategies among cetaceans and reveal how MHC transcriptional control continues to evolve. The combined MHC regulatory and coding data provide new genetic context for distinct vulnerability profiles between coastal and estuarine populations, which are key concerns for health and risk management.

## Introduction

The evolutionary arms race is most evident when considering disease-causing pathogens in mammals. A faster generation turnover in short-lived pathogens allows them to quickly adapt to the more slowly evolving defenses of their longer-lived mammalian hosts. The need to keep up with their pathogen counterparts is evidenced by the most quickly evolving portion of the vertebrate genome, the major histocompatibility complex (MHC), which encodes the proteins involved in triggering the adaptive immune response [1, 2]. While there are various hypotheses regarding the most influential driver of immunogenetic diversity (heterozygote advantage, rare allele advantage, fluctuating selection), the absence of genetic variation at MHC loci is generally considered to be a precarious state for any wild population [3–6]. Particularly, populations which are already at risk due to environmental deterioration may be easily jeopardized by a single disease outbreak. Two primary MHC gene classes are distinguished by whether they respond to internal cellular threats such as viruses (Class I) or external cellular threats by engulfing, degrading, and presenting pathogens from within their vicinity (Class II). All cells display Class I receptors, but only antigen presenting cells constitutively express Class II receptors in most mammals. Notable exceptions include two cetaceans; bottlenose dolphins and beluga whales exhibited continuous Class II expression on T lymphocytes [7, 8]. Class II receptors are heterodimeric molecules composed of the products from two separate genes; for example, the DQ receptor is encoded by *DQA* and *DQB*. Once a pathogen has been internalized and broken down within the cell, initiation of the adaptive immune response hinges on the ability of the receptor’s peptide binding region (PBR) to adequately bind the pathogen peptide fragment and present it to a T-cell receptor [9, 10]. The PBR is formed by a combination of the peptide products from the second exons from both *DQA* and *DQB*, thus variation at these exons has become a standard marker for predicting or understanding population responses to disease outbreaks [11–13].

More recently, increased understanding of the role of differential gene regulation has extended the focus to MHC promoter regions as well [14, 15]. Factors exist in the upstream control region (1–2 kb 5’ flanking sequence) which can alter expression, but the promoter proximal area (150–200 bp 5’ flanking sequence) has been more thoroughly characterized. Several motifs in this region are well-conserved among vertebrates: the S, X1/X2, and Y boxes [16]. These motifs must be bound by transcription factors RFX, CREB, ANK, NF-YA, YC, and YB which in turn are all bound to the Class II Transactivator (CIITA) [17]; all factors and CIITA must be in place to initiate transcription (S1 Fig). Although the transcription factors are always available, multiple strategies of regulation are at work in this process. In most mammals, CIITA is constitutively expressed in antigen presenting cells and is inducible in T helper cells. Four discrete promoter regions respond to different stimuli to instigate transcription of CIITA, which then acts as a master regulator to switch on all MHC Class II genes [18]. Another degree of control may be influenced through each MHC gene’s promoter proximal sequence; variation within or nearby the motifs may affect how well the motif/transcription factor/CIITA complex is formed. This may be the driving force behind findings of asymmetrical expression levels between Class II genes as well as between different alleles for the same gene [15, 19, 20]. A final consideration is the presence of gene-specific motifs, such as the T -box and NF-κB box which are found only in the *DQA* promoter [21].

Mammalian immune response pathways include a myriad of checkpoints and controls which can be applied via altered binding capacity for various pathogens or through the availability of receptors on the cell surface. An appropriate marker system should: a) Include the complete peptide binding region of both genes necessary to form the receptor molecule (i.e., *DQA* and *DQB* for DQ receptor), and b) Include the promoter regions responsible for regulating gene expression. The immune response of aquatic mammals is likely unique as these organisms experience waterborne, airborne, and even terrestrial pathogen insults [22, 23]. Harvell et al. reviews cases of marine mammal disease outbreaks which were likely initiated from a terrestrial host [24]. The taxonomic diversity of both host and pathogens is greater in marine environments and there is potential for more wide-spread ranges, making epidemiology modeling more challenging [25]. Determining the molecular basis of the initial triggers of the immune response in such species has become increasingly urgent, especially for coastal populations. Deteriorating environmental quality alongside increasing anthropogenic activities has been linked to growing health problems and emerging diseases in coastal and estuarine marine mammals [26–28].

**Fig 1:**
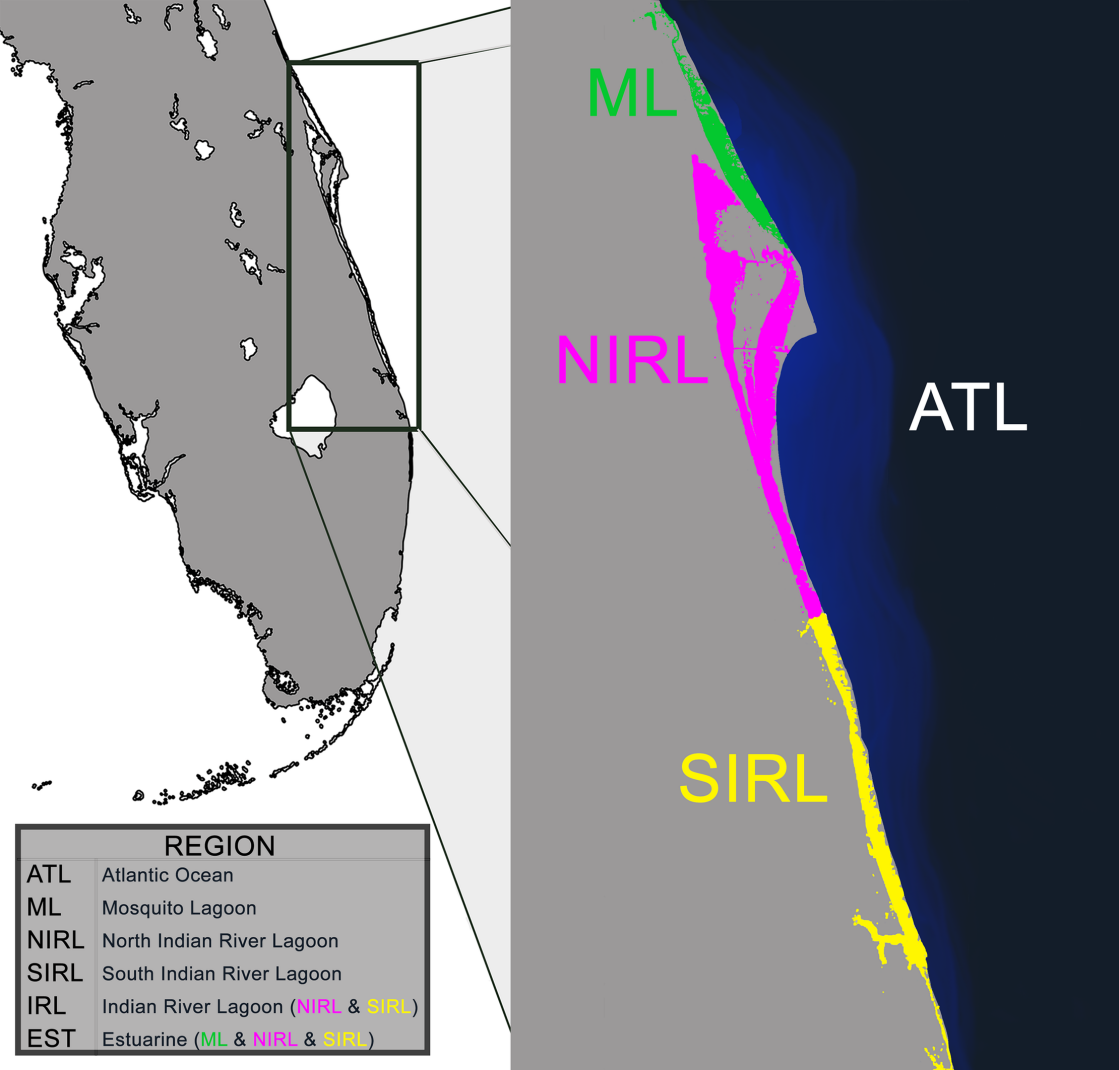
Geographic locations of *Tursiops truncatus* populations. Dolphins from Florida’s Indian River Lagoon Estuary System and Atlantic coast were divided into the following four sampling regions: **ATL** (Atlantic); **ML** (Mosquito Lagoon); **NIRL** (North Indian River Lagoon); **SIRL** (South Indian River Lagoon). Population analyses were performed on these four sample groups as well as on combinations of these groups to look for larger ecosystem differences (**IRL** = combination of NIRL and SIRL; **EST** = Estuarine, combination of ML, NIRL, SIRL).

Bottlenose dolphins reside in nearshore waters along Florida’s Atlantic coast as well as in a number of estuaries, bays, and lagoons, including the 256 km long Indian River Lagoon Estuary System (IRLES). Coastal populations, which may include migratory populations, have been found to be demographically and genetically distinct from a number of these embayed and lagoon populations [29–32], although some gene flow and movement has also been documented [32]. We hypothesize that the populations along Florida’s Atlantic coast likely experience a different pathogenic environment than the neighboring estuarine dolphins living where anthropogenic intrusion is more concentrated. The Indian River Lagoon (IRL), Mosquito Lagoon (ML), and Banana River are all part of the IRLES (Fig 1). Chemical contamination, high nutrient input, decreased salinity, loss of sea grass habitat, and eutrophication have all culminated in poor habitat quality in the IRL Estuary System. Disparity between population and even individual susceptibility to diseases has been documented for several cetaceans and is often linked to concentrations of immunosuppresive contaminants [28, 33, 34]. In 2013–2014 an unusual mortality event (UME) in Atlantic bottlenose dolphins extended down to the Florida Keys yet no affected individuals were found in the Gulf of Mexico [35, 36]. These data suggest that multiple factors may influence the spatial variation of disease transmission and affect neighboring populations to different degrees. Murdoch et al. [37] found that dolphins in the southern region of the IRL had a nearly 40-fold higher occurrence of lobomycosis and significant impairment of adaptive immunity in contrast to the northern IRL dolphins. The ML, the northernmost water body analyzed for this study, has only been connected to the IRL since the 1850’s following completion of the Haulover Canal [38] and has a single inlet access to the Atlantic Ocean. There may be different selection pressures acting on Estuarine versus Ocean populations of dolphin, between southern versus northern IRL dolphins, and between dolphins in the IRL and the ML.

Pathogen load information is difficult to obtain for cetaceans, thus MHC variation may be a useful alternative for studying their impact in association with environmental stressors. Furthermore, dolphins are susceptible to the same health hazards as humans including mercury, brevetoxin, and lobomycosis, such that they serve as a sentinel species to highlight concerns relevant to public health. Variable survival rates in dolphins during a harmful algal bloom has recently been linked to MHC diversity [39]. Multiple studies have been undertaken to examine class II exon 2 variation in dolphins and whales, yet most do not capture the entire PBR and none to date have typed the promoter region [39–46]. To develop a more comprehensive MHC marker system for cetaceans, the first objective was to characterize DQ proximal promoter regions and transcription factor binding sites across several families of cetaceans (Delpinidae, Mondontidae, Lipotidae, Physeteridae, Baleonopteridae) with available whole genome sequence (WGS) data. The second objective was to combine traditional and next generation sequencing methods for genotyping both the complete PBR and promoter regions of the DQ receptor molecule in *T*. *truncatus*. The third objective was to apply this marker system to investigate the evolutionary and demographic forces shaping dolphin MHC diversity in contrasting estuarine and marine habitats.

## Results

### 1) Promoter motif identification

**Fig 2:**
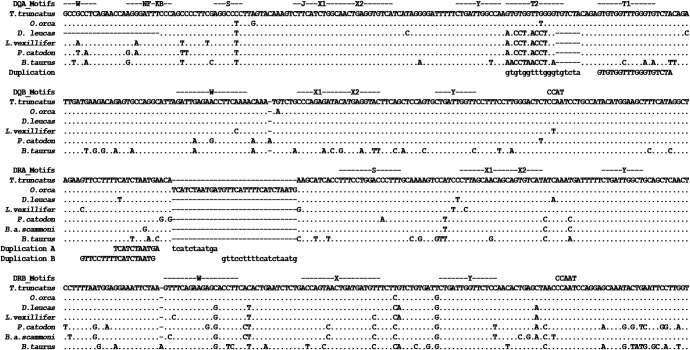
Conserved regulatory motifs of four MHC class II loci. The S(W), X, and Y boxes must be bound by transcription factors, which must then be bound by the class II transactivator for transcription to be initiated. Duplication (lowercase) and duplication source (uppercase) are indicated underneath *DQA* and *DRA*. Accession numbers are specified in Table 1; *D*. *leucas* data was derived from this study. In *DQA*, T2 refers to a T box motif duplication that is only present in *Tursiops truncatus* and *Orcinus orca*.

All MHC class II genes share evolutionarily conserved W/S, X1/X2, and Y boxes [47], but each gene may have unique motifs as well; *DQA* has an additional W box, and a NF-Kβ, and T-box. The promoter motifs for *DQA*, *DQB*, *DRA*, and *DRB* were located from each cetacean WGS dataset based on similarity to well-characterized model species (see Methods) [14, 48]. Potentially three separate duplications, ranging from 11 bp to 22 bp, were noted within two of the promoters of certain species (Fig 2). Two were found only in *Orcinus orca* at the *DRA* proximal promoter, both upstream of the S-box. The overlapping architecture results in three copies of an 11 bp motif (TCATCTAATGA) with variable spacer sequence resulting in a combined insertion of 30bp in length. Analysis of the WGS data also revealed a complete duplication of the 14 bp T-box in the reference dolphin genome. Alignment of the WGS of several cetaceans shows the duplication is also present in *O*. *orca* (diverged from dolphins 10.9 Mya), but absent from *Lipotes vexillifer* (25.4 Mya) and *Physeter catodon* (33.5 Mya) [49]. A 142 bp stretch beginning with the *DQA* W box and ending with the second T-box was queried against the complete current NCBI WGS database excluding *T*. *truncatus* (>30,000 WGS datasets). This search revealed that the only other full-length hit was to *O*. *orca*, suggesting that the duplicated T-box is a feature unique to a limited number of cetaceans. A 133 bp stretch encompassing the *DRA* duplications through the S-box were queried with no significant hits outside *O*. *orca* against the WGS database.

**Table 1:** Heterozygosity in *Tursiops truncatus* populations.

	Locus	n	O. Het	E. Het	P-value	s.d.
**Atlantic**	DQAE	22	0.955	0.866	0.228	0.003
	DQAP	20	0.700	0.706	0.489	0.006
	DQBE	15	1.000	0.883	0.910	0.001
	DQBP	22	0.636	0.726	0.644	0.004
**Mosquito Lagoon**	DQAE	22	0.636	0.738	0.189	0.003
	DQAP	18	0.500	0.557	0.413	0.003
	DQBE	18	0.667	0.797	0.111	0.003
	DQBP	19	0.211	0.738	0.000	0.000
**North Indian River Lagoon**	DQAE	18	0.444	0.589	0.198	0.003
	DQAP	17	0.294	0.273	1.000	0.000
	DQBE	17	0.529	0.643	0.503	0.004
	DQBP	15	0.200	0.632	0.001	0.000
**South Indian River Lagoon**	DQAE	30	0.633	0.599	0.778	0.003
	DQAP	30	0.467	0.414	0.789	0.004
	DQBE	29	0.793	0.699	0.849	0.003
	DQBP	28	0.250	0.706	0.000	0.000
**Indian River Lagoon Proper**	DQAE	48	0.563	0.591	0.972	0.001
	DQAP	47	0.404	0.370	1.000	0.000
	DQBE	46	0.696	0.679	0.936	0.003
	DQBP	43	0.233	0.686	0.000	0.000
**Estuarine**	DQAE	70	0.586	0.643	0.472	0.003
	DQAP	65	0.431	0.428	0.684	0.003
	DQBE	64	0.688	0.718	0.772	0.001
	DQBP	62	0.226	0.714	0.000	0.000

Expected heterozygosity did not differ from observed for *DQA* exon 2 (DQAE), *DQA* promoter (DQAP), or *DQB* exon 2 (DQBE) at any population. All populations except the Atlantic were significantly heterozygous deficient at *DQB* promoter (DQBP). Significant values are shaded.

### 2) Promoter Sanger sequencing

In-house Sanger sequencing was performed on *T*. *truncatus* (n = 10), *O*. *orca* (n = 1), and *Delphinapterus leucas* (n = 4) for the proximal promoter regions of *DQA*, *DQB*, *DRA*, and *DRB*. These data confirmed the WGS findings described above, showing a *DQA* T-box duplication in *T*. *truncatus* and *O*. *orca* and its absence in *D*. *leucas* (diverged from dolphins 18.4 Mya) [49]. The *DRA* duplications were confirmed in *O*. *orca* and absent from *T*. *truncatus* and *D*. *leucas* (see Fig 2). Unique sequences were uploaded to GenBank (KR067702—KR067727).

### 3) *DQA* PBR Sanger sequencing, cloning, and genotyping

A total of 66 individual dolphins were successfully sequenced for 529 base pairs of the *DQA* gene, including the entire 249 bp region of exon 2 which encodes the 82 aa PBR (S2 Fig). While 22 dolphins were homozygous, the remaining 44 had 18 potential heterozygous sites (double chromatogram peaks, S3 Fig). Ten of the heterozygous dolphins were selected such that all of the identified combinations of variable sites were represented. This subset was cloned alongside one of the homozygous dolphins, and 10–20 clones were picked and sequenced for each of the 11 dolphins. No more than two unique alleles were found for any one individual, indicating only one locus for *DQA* in *T*. *truncatus*. The homozygote/heterozygote identifications and the 18 variable sites were all confirmed, and 7 alleles were typed. These 7 alleles from the cloned dolphins were then used to call 57 out of the 66 dolphins that had been sanger sequenced. The last 9 (heterozygous) individuals were then called using the following assumption: Based on the frequency of known alleles, the remaining individuals likely had at least one previously typed allele, and potentially one new allele. Using parsimonious logic, three additional alleles were conservatively predicted. Due to the potential for rare alleles in highly polymorphic MHC loci, some alleles were identified in only one dolphin and they, along with the predicted alleles, could not be presented without caution. While cloning is an effective method for allele verification, this method is very cost-prohibitive and time consuming. However, the NGS data analyses described below resulted in the same final set of 10 alleles (7 from cloning and 3 predicted) from a larger sample set.

### 4) DQ promoter and PBR NGS sequencing

A more expansive sampling of *T*. *truncatus* (n = 95, see Methods) was then genotyped using Ion Torrent™ sequencing of the proximal promoter region and the exon 2 PBR for both the *DQA* and *DQB* genes. The statistics for the NGS run are as follows. For ease of description, the term sample refers to experimental sample instead of individual. One dolphin becomes four samples after PCR amplifying four distinct targets (*DQA* promoter, *DQA* PBR, *DQB* promoter, and *DQB* PBR). The Ion Torrent PGM run produced 5,215,027 reads, of which 2,938,180 were at least 350 bp. There were 2,349,931 reads with perfect barcode matches kept for downstream analyses. The average number of reads per sample is as follows: 8,644 for *DQA* promoter; 2,614 for *DQA* PBR; 9,097 for *DQB* promoter; and 3,144 for *DQB* PBR. Of the 400 samples sequenced (95 dolphins + 5 duplicates x 4 PCR targets), 360 produced usable data with at least 500 reads. The cutoff is based on the number of reads per meaningful barcode combinations. Several barcode combinations never made it into the pooled sample due to poor PCR amplification, yet those pairs were recovered during barcode sorting. This is likely due to PCR chimera formation of the pooled samples during library preparation prior to sequencing. Such chimera formation could easily distort the variant analysis by producing false unique alleles. The highest number of reads produced for an unused barcode combination was 489, thus providing an additional quality control benchmark. However, it is likely that chimeras were also formed during the first individual PCR reactions as well, as discussed below. Future work will incorporate the recommendations outlined by Lenz and Becker for reducing these artifacts [50]. After excluding low-read barcode pairs, the sample numbers are as follows: *DQA* PBR, n = 92; *DQA* promoter, n = 85; *DQB* PBR, n = 79; *DQB* promoter, n = 84 (as well as the quality control samples). The final data set used for the analyses included 2,332,297 sequence reads. Although there are additional errors described below, the reads still produced useful information as long as the appropriate variant identification algorithm was applied. After parsing for a minimum of 500 reads per sample, there was no set minimum read count for allele designation outside of the default Phred-scaled logarithm of odds (-q 40) applied by SAMTools for calling heterozygotes versus homozygotes [51].

Along with the PCR chimera formation, several sources for potential error were identified using multiple controls. Five dolphins were run twice by using different barcode pairs in separate PCR reactions for all four PCR targets. This served as an internal control of method precision. Four of the samples returned identical variant calls for all four targets while the fifth sample was confirmed in three targets. Low read number at the fourth target led to its exclusion prior to variant calling. Sixty-six samples typed for *DQA* PBR using traditional methods (see above) served as an external control; sixty-five were in agreement with this study, but one sample was excluded due to low sequence read number. Finally, the two separate variant analyses (UnifiedGenotyper and SAMTools) each had certain advantages over the other which emphasize the need for multiple approaches and full understanding of parameters for capturing all variable sites. The following statistics are for the percent agreement between the two analyses and the total number of variant calls from each dataset after excluding low-read samples: DQA promoter, 100% of 267 calls; DQA PBR, 95% of 907 calls; DQB promoter, 97% of 407 calls; and DQB PBR, 97% of 1703 calls. Calls which were not in agreement were selected for further analysis of the raw sequence data to determine the cause for each incongruity. For an in-depth comparison of variant calling methods, refer to Hwang et al. [52]. Through a combination of the previously typed samples and both analyses, it was possible to identify five primary sources of error as follows. **1)** Sequencing error: While most samples had clean sequences (high identity between reads), the few which had lower identity had false positive calls by UnifiedGenotyper. SAMTools is much more sensitive to allele ratios and thus had a higher threshhold which prevented false calls from sequencing errors. Sanger sequences confirmed the SAMTools calls in these cases (S4 Fig). **2)** PCR chimeras: On the flip side, SAMTools’ sensitivity to allele ratios causes it to ignore variable sites which did not occur in nearly 50 or 100% of the reads. PCR chimeras can throw off the ratios by creating three dominant alleles in a sample, one of which is clearly a combination of the two true alleles. This common PCR artifact might be overlooked or lead to large amounts of unusable data if analyses are restricted by a minimum number of reads per called allele; however, UnifiedGenotyper correctly called these samples, as confirmed by Sanger sequencing (S5 Fig). **3)** TMAP misalignment: This issue appeared to arise from non-random sequencing errors in a single orientation. Single base deletions would occur in only the forward (or only the reverse) reads, and TMAP incorrectly compensated by adding extra indels. The resulting misalignment affected the variant analyses differently depending on how many reads were present from each sequencing direction. Once this was flagged as a problem site in one sample, the remaining alignments were verified manually and sites were confirmed through Sanger sequencing (S6 Fig). **4)** Tri-allelic variable sites: *DQB* exon alleles had five sites which had three possible alleles. SAMTools is not written to handle these sites, but UnifiedGenotyper correctly calls them. However, the downstream analyses did not phase these genotypes and thus the alignments were checked manually to determine phasing at tri-allelic sites (S7 Fig). **5)** Homopolymers: Two cases of variants falling within homopolymer stretches were ignored by UnifiedGenotyper and correctly called by SAMTools. The variant either created or elongated a homopolymer (ATA to AAA and T_4_AT_2_ to T_7_). Even in homozygous samples UnifiedGenotyper would not call a variable site within a homopolymer (S8 Fig). The errors described above were all first flagged as conflicting variable site calls between UnifiedGenotyper and SAMtools. Manual review of the sample raw data was performed for each instance to identify the cause, and Sanger verification was performed as needed. This allowed the strengths of each algorithm to be employed rather than purging data.

#### Alleles

Alleles were named using the proposed method from Klein et al. [53] and sequence data have been uploaded to public repositories (NGS raw data on Dryad, https://doi.org/10.5061/dryad.r36sg; Sanger promoter data on GenBank, (KR067702-KR067727); and final called alleles on GenBank (MG211337-MG211369). The *DQA* promoter was typed for 6 alleles with 4 variable sites, and *DQB* promoter had 7 alleles with 9 variable sites (S9 and S10 Figs). Two *DQA* variable sites occurred within regulatory motifs; a C/T variant is present in the S box and a G/T variant is present in the 5’ T-box. All the *DQB* variants arose outside the motifs. Both *DQA* and *DQB* PBR had 10 alleles, the *DQA* PBR had 18 variable sites (S11 Fig) while *DQB* PBR had 53 (S12 Fig), 5 of which were tri-allelic (S7 Fig). The same alleles were identified for *DQA* PBR using Sanger and NGS, although the primers and amplicon lengths differ (Refer to Methods).

### 5) Population and cross-taxa analyses

Neighbor Joining Trees for *DQA* promoter, *DQA* PBR, *DQB* promoter and *DQB* PBR can be found in S13 Fig. These depict only the similarity between the alleles and should not be viewed as a phylogeny; they were not used for analyses, only for illustrative purposes. The following analyses examined the different evolutionary forces acting upon MHC class II loci in *T*. *truncatus*.

**Fig 3:**
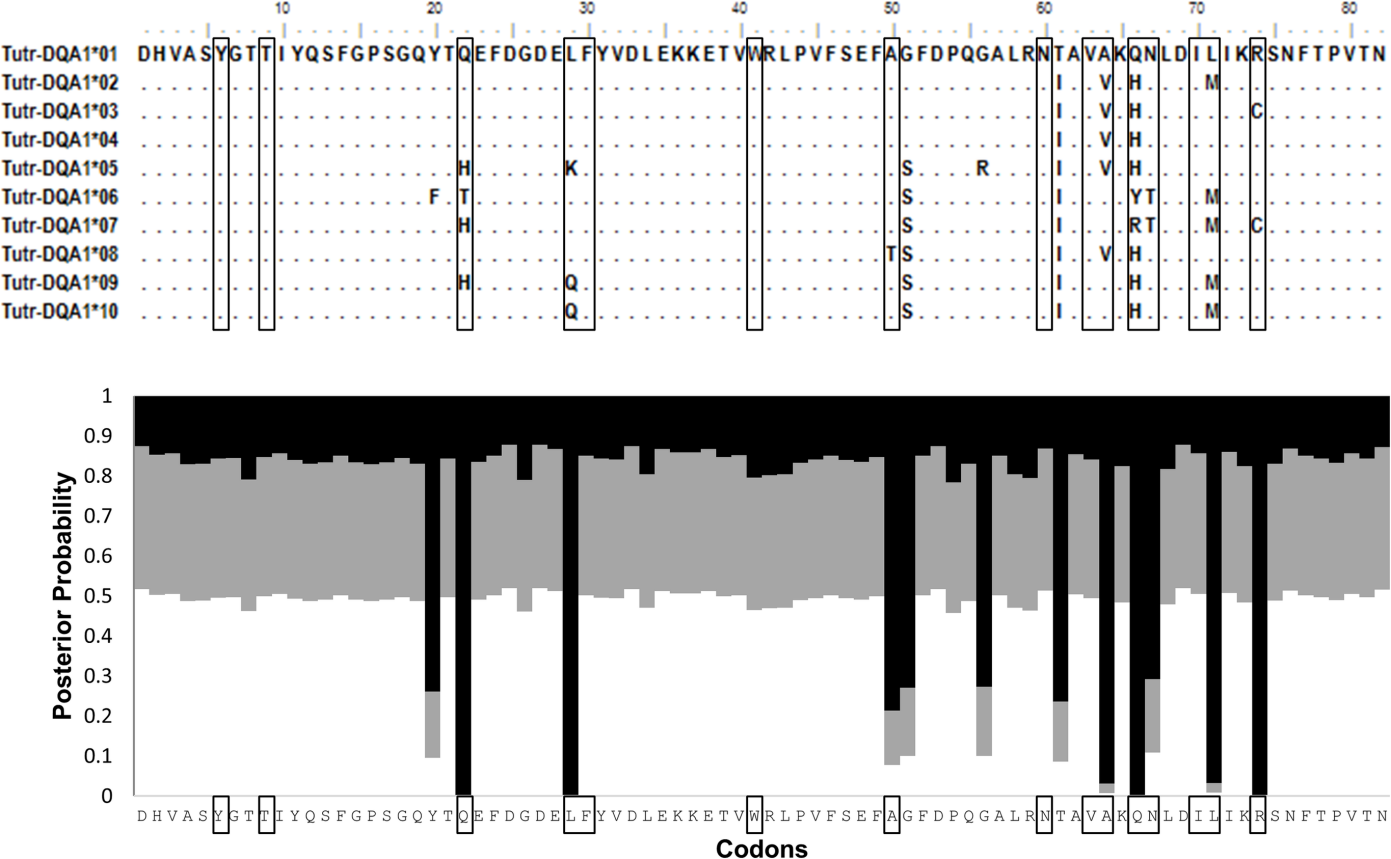
Protein sequences for *DQA* exon 2. Top: Fifteen boxed amino acids form the peptide binding pockets in *Tursiops truncatus*. Bottom: All twelve variable sites are likely under positive selection, as indicated from Bayes Empirical Bayes probabilities on dN/dS (w_0_<1, white; w_1_ = 1, gray; w_2_>1, black).

**Fig 4:**
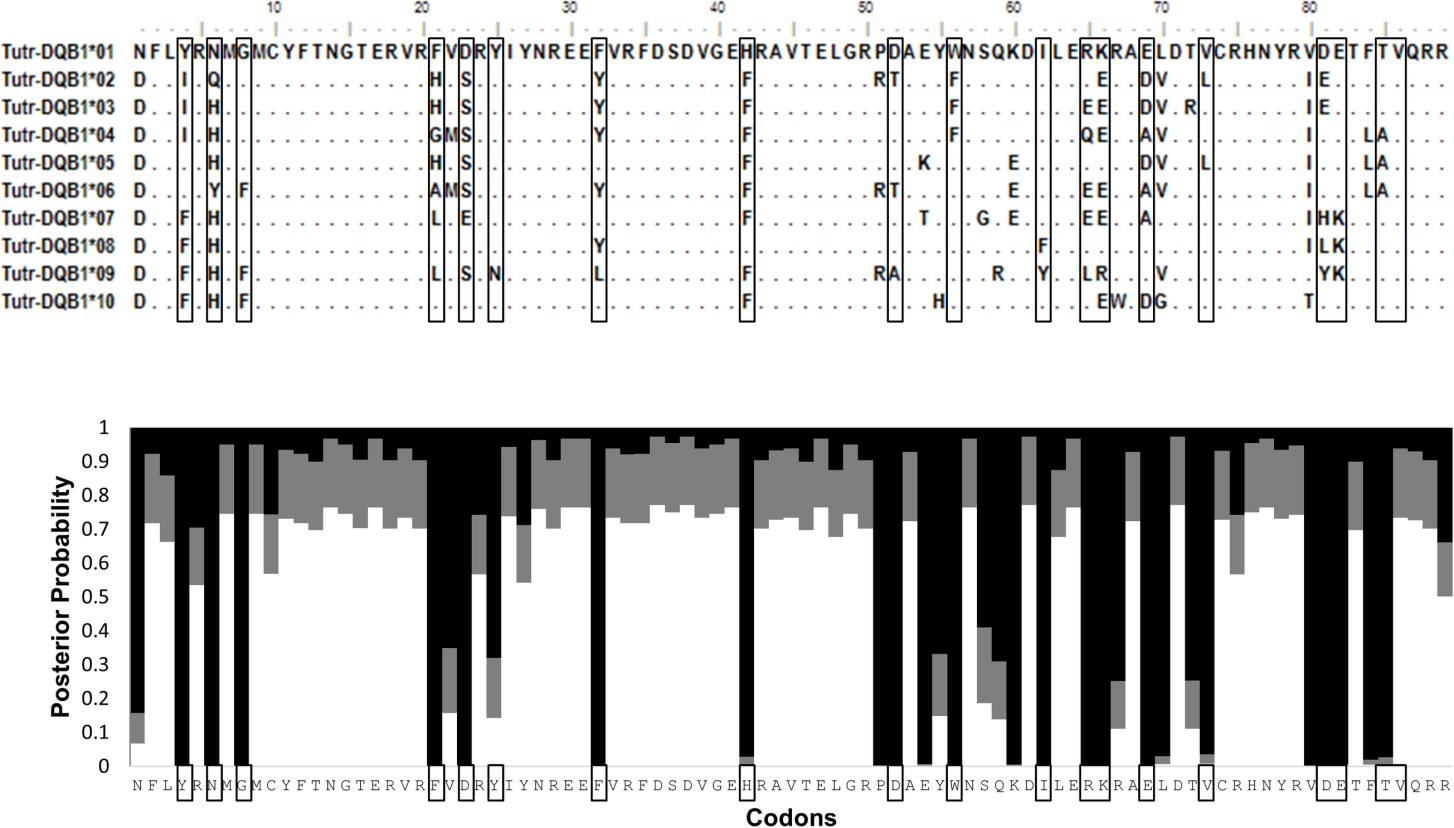
Protein sequences for *DQB* exon 2. Top: Nineteen boxed amino acids form the peptide binding pockets in *Tursiops truncatus*. Bottom: All thirty-one variable sites are likely under positive selection, as indicated from Bayes Empirical Bayes probabilities on dN/dS (w_0_<1, white; w_1_ = 1, gray; w_2_>1, black).

To assess mutations within the PBR, amino acid variation within exon 2 of *DQA* and *DQB* was examined in relation to the predicted antigen binding pockets described in the Methods. Having successfully captured the complete 82 aa for exon 2 using two distinct sequencing approaches, this study thoroughly assessed potential variation at all residues across the *DQA* binding pockets. Of 12 variable aa sites, 8 occur within the 15 aa forming the 3 pockets (Fig 3); thus a significantly high occurrence of amino acid variation was found within functional peptide binding sites (G = 13.487, P<0.001). As with *DQA*, a significantly high proportion of the DQB exon’s variable amino acid sites (18 out of 31) occur within the 19 aa forming 5 binding pockets (Fig 4, G = 19.700, P<0.001).

The transition/transversion bias (R) was estimated at 0.79 for *DQA* coding region (max log likehood -509.942) and 0.64 (-902.400) for *DQB*, indicating that these substitution types do not occur in either dataset according to their probability (R = 0.5). Transversions should occur twice as often as transitions, yet that is often not the case [54]. Codon usage analysis suggested that *DQB* exon 2 may have strong bias with a CBI of 0.75 (scale from no bias at 0.0 to high bias at 1.0) and Nc of 29.088 (scale from high bias at 20 to no bias at 61). *DQA* results suggest a more random codon usage with CBI 0.398 and Nc 58.921. Additionally, *DQB* PBR had higher GC content (60%) than *DQA* (49%). All of these factors can affect mutation rates and thus deviations from the predicted probabilities will skew analyses which do not account for them.

To test for evidence of selection in both DQ coding regions, the dN:dS ratio (w), was calculated using a maximum likelihood approach. All equally parsimonious trees within the allele networks and the different runmodes in CodeML yielded similar results. The combined ratios across all codons were >1, suggesting positive selection is shaping the genotypic variation observed in both *DQA* (w = 1.840) and *DQB* (w = 2.754) PBRs. Bayes Empirical Bayes (BEB) probabilities for individual codons across entire exon 2 regions also suggest that all the variable amino acid sites in DQA and DQB are under positive selection. This includes all except one of the 19 binding pocket aa sites in DQB, along with 8 out of 15 of the binding pocket sites in DQA (Figs 3 and 4).

The following micro-geographic scale was analyzed in four sample groups: Atlantic (ATL), Mosquito Lagoon (ML), North Indian River Lagoon (NIRL), and South Indian River Lagoon (SIRL). Analyses on a larger *Water Body* scale were performed by combining North and South IRL (IRL), and then a macro-geographic *Ecosystem* scale was analyzed by combining all three estuarine sample groups (EST, see Fig 1). Observed heterozygosity did not differ significantly from expected heterozygosity within any of these scales for three of the four loci: *DQA* promoter, *DQA* PBR, and *DQB* PBR. The one exception was that at all scales except the Atlantic, *DQB* promoter was noted to be heterozygote deficient (Table 1, p<0.0005). Geographic differentiation was observed at the DQ loci at a number of spatial scales. With the exception of a single DQB PBR allele, all DQ promoter and PBR alleles (Table 2) were identified in the Atlantic samples (97% of 33). Allelic diversity was moderate in Mosquito Lagoon (70%) and lower in both North and South IRL (52%). A summary of allelic representation by geographic region can be found in Table 2. At the macro-geographic *Ecosystem* scale, the Atlantic was substantially differentiated from the combined Estuarine habitats at each locus (*F*_st_ = 0.084–0.141, p = 0.00000–0.00218) and all loci combined (*F*_st_ = 0.084, p<0.001, Table 3). At the mid-level *Water Body* scale, the Atlantic was significantly differentiated from the IRL (*F*_st all loci_ = 0.113, p<0.001) but not from the ML which was intermediate between the Atlantic and IRL (*F*_st all loci_ = 0.022–0.027, p>0.05). At the micro-geographic scale, no differentiation was found within the IRL (*F*_st all loci_ = -0.015, Table 3).

**Table 2:** Allelic frequencies by geographic region.

*DQA* Alleles	ATL	ML	NIRL	SIRL	*DQB* Alleles	ATL	ML	NIRL	SIRL
Tutr-DQAP1*01	4	6	—	—	Tutr-DQBP*01	3	4	6	6
Tutr-DQAP1*02	12	5	2	14	Tutr-DQBP*02	6	15	17	26
Tutr-DQAP1*03	2	—	2	2	Tutr-DQBP*03	1	2	3	14
Tutr-DQAP1*04	3	2	1	—	Tutr-DQBP*04	1	—	—	—
Tutr-DQAP1*05	1	—	—	—	Tutr-DQBP*05	15	12	4	4
Tutr-DQAP1*06	18	23	29	44	Tutr-DQBP*06	17	4	—	6
					Tutr-DQBP*07	1	1	—	—
Tutr-DQA1*01	7	16	15	27	Tutr-DQB1*01	6	2	—	4
Tutr-DQA1*02	10	15	18	27	Tutr-DQB1*02	—	1	—	—
Tutr-DQA1*03	2	1	2	2	Tutr-DQB1*03	2	—	—	—
Tutr-DQA1*04	8	2	—	4	Tutr-DQB1*04	3	11	16	26
Tutr-DQA1*05	5	2	1	—	Tutr-DQB1*05	1	—	—	—
Tutr-DQA1*06	2	—	—	—	Tutr-DQB1*06	5	2	1	—
Tutr-DQA1*07	1	1	—	—	Tutr-DQB1*07	6	6	—	—
Tutr-DQA1*08	7	7	—	—	Tutr-DQB1*08	1	2	2	10
Tutr-DQA1*09	1	—	—	—	Tutr-DQB1*09	2	1	2	2
Tutr-DQA1*10	1	—	—	—	Tutr-DQB1*10	4	11	13	16

The frequency of *DQA* (left) and *DQB* (right) promoter (top) and exon 2 (bottom) alleles are shown. The geographic regions from Fig 1 are Atlantic (ATL), Mosquito Lagoon (ML), North Indian River Lagoon (NIRL), and South Indian River Lagoon (SIRL). Alleles found only in ATL and ML are shaded.

**Table 3:** Population pairwise FSTs.

	ATL	ML	NIRL	SIRL	IRL	EST
**ATL**	0.000					
**ML**	0.027	0.000				
**NIRL**	0.104	0.017	0.000			
**SIRL**	0.102	0.016	-0.015	0.000		
**IRL**	0.113	0.022	-0.016	-0.013	0.000	
**EST**	0.084	0.002	-0.010	-0.008	-0.005	0.000

Pairwise differences were calculated using 10100 permutations and p<0.05 (shaded) in a combined analysis of *DQA* and *DQB* promoter and peptide binding region alleles in *Tursiops truncatus*. Population designations from Fig 1 are: Atlantic (ATL), Mosquito Lagoon (ML), North Indian River Lagoon (NIRL), South Indian River Lagoon (SIRL), NIRL and SIRL (IRL), ML and IRL (EST).

**Fig 5:**
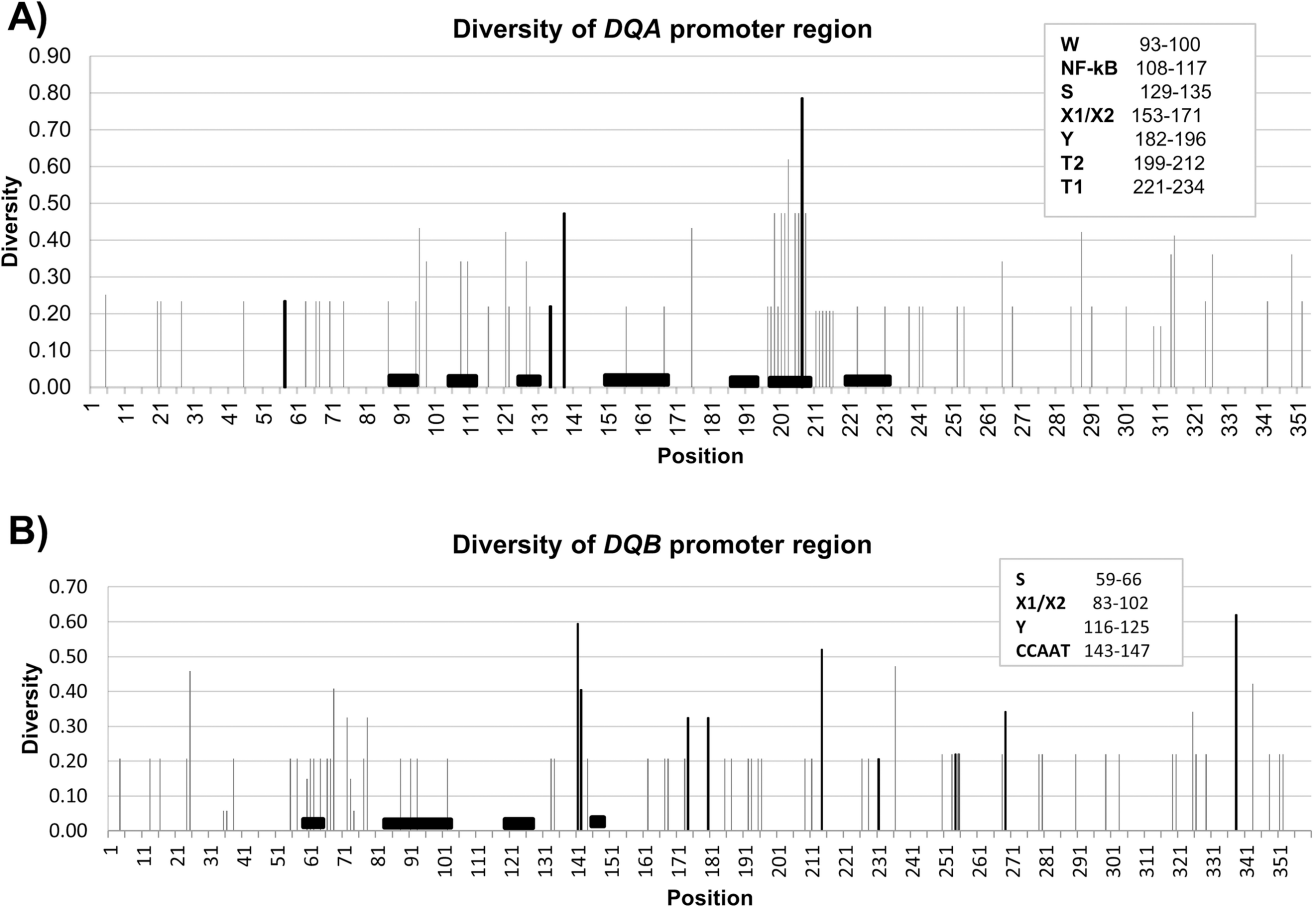
Shannon entropy for DQ promoter regions. Shannon entropy was measured across the promoter regions for *the Tursiops truncatus* alleles described in text and a sequence from each of *Orcinus orca*, *Delphinapterus leucas*, *Lipotes vexillifer*, *Physeter catodon*, and *Bos taurus*. Transcription factor binding motifs are described in the legend and illustrated by black boxes. Variable sites within *T*. *truncatus* are indicated with bold lines. Note that most of the variation displayed for *DQA* T2 is due to its absence in all but dolphins and orcas.

Lastly, Shannon entropy was used to characterize the variability of the promoter regions. This diversity index is actually a measure of uncertainty and is intended only to provide a rough estimate of which sites are conserved and which are variable [55]. Within *T*. *truncatus*, there was no variation inside the transcription factor binding motifs for *DQB*, although two variable sites were noted inside *DQA* motifs (Fig 5). Higher diversity can be seen across additional taxa, with no apparent bias for or against motifs. The high level of variation observed at T2 in *DQA* was likely from a single event where the first T-box was duplicated and replaced a short sequence (*T*. *truncatus* and *O*. *orca*, Fig 2).

## Discussion

The genetic diversity of MHC has long been applied to population studies as a measure of health, but data based on a single exon from a single gene cannot reliably predict how a population will fight a disease outbreak. The complex expression pathway from genotype to immune response is so convoluted that MHC studies may often be over-simplified and draw irrelevant conclusions. A few cetacean studies have analyzed both *DQA* and *DQB* [39, 40, 42, 43], yet none to date have captured the entire *DQA* exon 2 coding region (Fig 3). Furthermore, no cetacean studies have yet examined the proximal promoter regions which have been recognized as having a pivotal role in the efficiency of immune response initiation [19, 56]. This study has characterized the promoter regions for *DQA*, *DQB*, *DRA*, and *DRB* across four cetacean families. The promoter regions and peptide binding regions of *DQA* and *DQB* in *Tursiops truncatus* were genotyped across two distinct ecosystems (ocean versus estuarine), of which the latter was further subdivided by separate waterbodies (Mosquito Lagoon versus Indian River Lagoon) or subregions (North versus South Indian River Lagoon), and revealed substantial genetic variation, including some substitutions within regulatory motifs of the promoters.

Alignment of the MHC class II proximal promoter regions across bottlenose dolphin, killer whale, beluga whale, Chinese river dolphin, and sperm whale highlighted some unexpected findings. Several short (11 to 22 bp) duplications were identified, including an intact copy of a 14 bp transcription factor binding site, in promoter regions that have been highly conserved across vertebrates in motif composition and spacing (Harton and Ting 2000). The extra *DQA* T-box copy was identified in *T*. *tursiops* and *O*. *orca* online WGS data and both were confirmed through in-house Sanger sequencing. Likewise, *O*. *orca* DRA was confirmed to carry an 11 bp sequence that occurs three times in contrast to once in the other cetaceans that were analyzed. The *DQA* promoter variant is of great interest because the duplication completely replicates a regulatory motif. The additional T-box arises closer to the Y-box than the original and appears to have replaced a short DNA segment; the full duplicated length is 18 bp, which has replaced and extended the flanking region between the Y and original T-box (Fig 2). Although there is little published on its regulatory effects, Morzycka-Wrobleska [20] suggests the T-box may decrease *DQA* expression in mice. Perhaps this occurs through stoichiometric interference with the CIITA/transcription factor/S-X-Y box complex formation; both motif sequence and spacing have been highly conserved and shown to affect transcription [57]. The duplicate T-box may exert a stronger influence due to its closer proximity to the S-box. A polymorphic site was found in the duplicated motif (Fig 5), which suggests that whatever factor(s) binds to the T-box may not bind equally to the two motifs now present. The T-box was first described as having similarity to a TNF-α response element [21]; however, data are insufficient regarding the function of the original T-box and thus potential adaptive roles for the duplicate cannot yet be surmised. The apparent fixation of such a significant deviation from the evolutionarily conserved norm in both *T*. *tursiops* and *O*. *orca* suggests that it provides some advantage. It may be worth noting that regulatory changes in only one of the A or B genes is required to lower the production rate of the receptor molecule. The MHC expression pathway is already highly regulated, but fine-tune controls for each locus may be imperative for promoting a healthy response without instigating an autoimmune reaction. It is not yet clear what genetic factor may be responsible for the constitutive T-cell activation identified in both dolphin and beluga species [7, 8]. Future efforts will continue to focus on this trait as well as elucidating the effects of both single and double T-box promoter loci in cetacean species.

Previous dolphin MHC work includes Arbanasic et al. [40] and Heimeier et al. [43], who amplified the full DQA exon. However, the internal placement of the forward primer could result in allelic dropout if single nucleotide polymorphisms (SNPs) occur within the priming region. All alleles described here differed from the Adriatic Sea dolphin alleles (Arbanasic) by three positions within their DQAex2F priming site. Their low (50°C) annealing temperature may have allowed for nonspecific primer binding, thus there are three potential *DQA* alleles shared between the data sets if the forward priming site is excluded. Beyond the binding pockets, there is additional information within the coding region such as a structural intra-chain salt bridge encoded within the DQAex2F primer binding site [58]. For these reasons new primers were designed for this study which lie outside the exon boundaries. While these primers capture a larger number of informative sites, they complicate the comparison of our alleles to other studies. Trimming all data down to the smallest common region (227 bp in *DQA*, 169 bp in DQB) reveals several ‘shared’ alleles from different geographic locations (S1 and S2 Tables), which could indicate conservation of important ancestral alleles or convergent evolution under similar pathogenic pressures [59]. However, all alleles described here have been uploaded to Genbank as unique due to their greater length (SNPs may occur outside the common region to previously described shorter alleles). Sanger sequencing was first performed for a subset of dolphins at *DQA* exon 2, with cloning verification. This data was used to groundtruth the larger NGS dataset from Ion Torrent PGM and highlighted several sources of error (S4–S8 Figs). Each sequencing approach and variant analysis has limitations, all of which are more apparent when using a combined methods approach. The resulting data identified a similar number of alleles across *DQA* and *DQB*, although the variability within alleles was higher for *DQB* than for *DQA* (S9–S11 Figs). Furthermore, the dN:dS ratio was higher for *DQB* PBR (2.754) than for *DQA* (1.840), although both are indicative of positive selection. The only deviation from expected heterozygosity was identified as a deficiency for the *DQB* promoter in all lagoon dolphin populations (Table 1); as a regulatory region, promoter diversity is likely subject to a different kind of selection than the PBR. Because they are noncoding sequences, fewer analyses are available for examining how selection might act on promoters beyond finding methods to characterize variation (Fig 5). In contrast, the coding region holds additional clues to how selection may act when considering the location of the residues in antigen binding pockets (Figs 3 and 4). In *DQA* and even more strikingly in *DQB*, the data demonstrates an advantage for diversity at specific codons. The majority of nonsynonymous changes occur in these pockets.

Positive selection is one of the most identifiable characteristics of MHC, and accounts for increased levels of polymorphism within the PBR [60, 61]. However, the commonly used Nei-Gojobori (NG) method with Jukes-Cantor correction assumes that transitions and transversions occur equally and that there is no codon bias [62, 63]. Transversions are more rare than transitions (*DQA* R = 0.79, *DQB* R = 0.64), which could lead to an inflated dS. Codon Usage Bias (CBI) and the effective number of codons (Nc) address the occurrence of unequal codon frequencies for a gene caused by selection, another factor which can interfere with dS. The CBI (0.75) and Nc (29.088) for *DQB* clearly showed deviations which could disrupt dN/dS calculations. Thus, a maximum likelihood method was used to estimate dN/dS while incorporating parameters for transition/transversion rates and codon bias. Although the ML values were lower than the YN estimates (1.8 vs 2.2 for *DQA*, 2.8 vs 4.2 for *DQB*), these values are likely more realistic and in combination with observed amino acid changes in binding pockets they offer compelling evidence for positive selection on DQ loci. The differences noted between *DQA* (Fig 3) and *DQB* (Fig 4) could result from higher selection pressures for variability in *DQB*. Despite the same number of alleles (10) for both PBRs, there are more sites under positive selection within DQB.

The specific selection pressures faced by bottlenose dolphins may vary by habitat (Fig 1). Combined calculations of differentiation (F_ST_) for all loci between Atlantic and Indian River Lagoon (IRL) populations showed significant differentiation (p<0.05, Table 3), while the Mosquito Lagoon (ML) population may share similarity with both IRL and Atlantic individuals. Further single-locus analyses showed the same pattern for *DQA*, yet Atlantic and ML populations appear to be significantly differentiated for *DQB*. The only significant F_ST_ for lagoon populations was between ML and North IRL for *DQA* promoter region. These findings are in line with a study on neutral markers which indicate that IRL dolphins are genetically distinct from Atlantic dolphins, while Mosquito Lagoon may host individuals from both habitats and thus this environment facilitates limited gene flow [32]. There were five alleles which were found both in Atlantic and ML populations but not the IRL (shaded, Table 2). The IRL has a smaller estimated population size ranging from 206 to 1,316, which is considerably lower than the Central Florida Coastal Stock report of 4,895 [64]. The lower estuarine diversity could result from genetic drift or from a more specialized adaptation to the local pathogenic community. In either case, it appears that the genetic framework for population immune response differs between the two environments and they may not respond similarly to emerging diseases or epidemics.

In the last twenty years, epizootics from viral infections have been responsible for the loss of thousands of marine mammals [65, 66]. Marine mammals are also susceptible to many non-viral threats, such as microparasites *Brucella*, *Toxoplasma gondii*, and *Lacazia loboi* along with other opportunistic bacteria and fungi [28]. Pathogen loads are difficult to obtain for cetaceans with limited access to individuals because of their marine habitat, intermittent sighting, and often decayed remains from stranding. The MHC marker system can predict how vulnerable current populations might be to future threats. Characterization of the genetic sequence can be taken to the next level to generate the peptide binding region of a MHC receptor molecule and determine the specific pathogens to which it can effectively bind. The capability to obtain DNA and employ it to create specific pathogenic susceptibility reports would be beyond valuable for handling epidemics in real-time. As this study has established a case for differential MHC allelic diversity between estuarine and coastal populations, collaborators are now using these data for identifying specific cetacean pathogens from binding affinities to several of the DQ alleles described here [67].

To summarize, higher allelic diversity in the larger population of coastal Atlantic dolphins compared to their estuarine neighbors highlighted immunogenetic differences at the macrogeographic level. These findings suggest that coastal and estuarine populations may be affected differentially by a disease outbreak, which prompted the recently published study described above to identify specific pathogenic binding affinities to different MHC alleles [67]. The characterization of MHC Class II promoter regions across different cetacean families revealed novel structural variation which provide a valuable addition to our understanding of immunogenetic evolution. The history of MHC evolution is written in gene duplication events [68]. While evidence for these larger-scale events is abundant, it is unclear how often promoter motif duplications occur; perhaps this is a frequent occurrence as well. Viville [69] and Basta [70] have both noted the occurrence of S motif duplications in class II promoters of mice and humans. Motif duplication events have the potential to modify the regulatory pathways for pathogen defenses. There is evidence that the S-box arose as a copy of the X-box [71]; both motifs are now canonical transcriptional regulators for an entire class of immune response genes. The relatively new duplication of another motif described here might play a pivotal role in determining alternative immunogenetic strategies for a group of species subjected to an inimitable environment of land, sea, and airborne pathogens.

## Materials and methods

### Sample collection and DNA extraction

The study region includes Mosquito Lagoon, and Banana and Indian River, which form the 200km long Indian River Lagoon (IRL) along Florida’s east coast (29.07°N, 80.92°W to 26.94°N, 80.08°W), along with the adjacent Florida Atlantic coastal regions (Fig 1). Tissue samples were collected from dolphin strandings, live capture studies [72], or via remote biopsy darting (National Oceanic and Atmospheric Administration permit no. 998-178-00) following all welfare procedures as detailed in the permit. Tissues were preserved in 20% dimethyl sulfoxide saturated with sodium chloride [73]. These samples were extracted as part of a related study on population and community structure of IRL dolphins involving detailed mitochondrial and microsatellite analysis [32]. The microsatellite analysis ensured that no individual was sampled multiple times. Samples were chosen to represent distinct locations where geographic separation and genetic discontinuities [32], ecosystem differences, and differences in disease prevalence [37] may reflect different pathogenic insults and thus immune responses. A total of 95 dolphins were analyzed from the following four geographic locations: Mosquito Lagoon (ML, n = 24), the North IRL (NIRL, n = 18), the South IRL (SIRL, n = 30), and the coastal Atlantic Ocean (ATL, n = 23). Population analyses were performed among those four sample groups, as well as using larger groups created by combining the north and south IRL groups (referred to simply as IRL, n = 48) and then combining the IRL with ML to encompass all estuarine sample groups (EST, n = 72). Only samples with a known mitochondrial haplotype were used. DNA extraction was carried out by Rodgers [32]; tissue samples were extracted using a sodium chloride protocol and total DNA quantified on a Nanodrop.

In order to improve the MHC marker system for cetaceans, the proximal promoters and complete peptide binding regions for *DQA* and *DQB* were characterized then genotyped from distinct populations of bottlenose dolphins. The remaining methods and results are divided as follows: **1)** Acquire MHC II GenBank mRNA and WGS sequence data from all available cetaceans, and identify exon 2 (PBR) and proximal promoter regions with transcription factor binding sites. **2)** Develop primers to confirm promoter regions in dolphin, orca, and beluga samples. **3)** Develop primers and Sanger sequence a subset of dolphins at *DQA* PBR with cloning confirmation to groundtruth next generation sequencing (NGS) methods. **4)** Develop primers and use NGS to sequence four areas of interest (*DQA* promoter, *DQA* PBR, *DQB* promoter, *DQB* PBR) in estuarine and coastal dolphins, and confirm findings against Sanger data. **5)** Perform analyses to identify potential effects of selection, drift, mutation, and gene flow at the population level in *Tursiops* and also across Cetacea.

### 1) *DQA/DQB* exons and promoter motif identification

Lack of a high-coverage reference genome in the past may have hindered the development of more informative primers, as many studies have relied on amplicons which do not fully capture the entire exon 2. However, there are now sufficient WGS and other sequence data available. In order to completely capture the PBR and the promoter regions for the DQ receptor molecule, GenBank was first searched for cetacean mRNA data. These sequences were then queried against each species’ WGS dataset using NCBI BLASTn [74] (see Table 4 for accession numbers). Dolphin mRNA was used as a query for any species with WGS but no mRNA data available (*O*. *orca* DQB and DRB, *L*. *vexillifer* DRB, *Balaenoptera acutorostrata scammoni* all loci). The mRNA and WGS sequences were aligned using BioEdit Sequence Alignment Editor 7.0.8.0 [75] to determine exon/intron boundaries. GENSCAN 1.0 [76] was run locally to confirm exon boundaries. Both *DQA* and *DQB* exons have incomplete codons; a codon begins at the end of exon 1 and is continued in exon 2, thus the partial codon was removed from exon 2 prior to population analyses (see step 5 below). The proximal promoter regions were located from the 200 bp region upstream of the transcription start site for exon 1. Key regulatory motifs were identified including the S, X1/X2 and Y boxes at both loci, the *DQA* W, NF-kβ, and T-box, and the *DQB* TTAA box. The highly conserved sequence and spacing of regulatory regions in MHC proximal promoters across mammals allowed identification of the motifs following the published data from other species [20, 77–79]. An additional cetacean, *Balaenoptera acutorostrata scammoni*, was also queried but significant matches were not found for *DQA* or *DQB* from the current dataset. Finally, the *DRA* and *DRB* proximal promoters were also identified and characterized as well as they have, to our knowledge, never been analyzed in cetaceans either.

**Table 4:** Reference sequences.

	Species	*DQA*	*DQB*	*DRA*	*DRB*
**mRNA**	*T*. *truncatus*	XM_004317915.2	XM_004317917.1	XM_004325137.1	EF017817.1
	*O*. *orca*	XM_004285618.1		XM_004285623.1	
	*L*. *vexillifer*	XM_007451935.1	AY177150.1	XM_007459506.1	
	*P*. *catodon*	XM_007123824.1	AB164208.1	FM986352.1	DQ354688.1
	*B*. *s*. *acutorostrata*				
**WGS**	*T*. *truncatus*	ABRN02290675.1	ABRN02290676.1	ABRN02517189.1	ABRN02517193.1
	*O*. *orca*	ANOL02074677.1	ANOL02074677.1	ANOL02074684.1	ANOL02074682.1
	*L*. *vexillifer*	AUPI01043388.1	AUPI01043385.1	AUPI01086382.1	AUPI01086381.1
	*P*. *catodon*	AWZP01083952.1	AWZP01083953.1	AWZP01060543.1	AWZP01091835.1
	*B*. *s*. *acutorostrata*			ATDI01093657.1	ATDI01040007.1
	*B*. *taurus*	NW_003104552.1	NW_003104554.1	NW_003104553.1	NW_003104553.1

Reference mRNA sequences were downloaded from GenBank and used for primer design and to query WGS for complete contigs; the top WGS hits are shown for each locus. Blank cells indicate no data available or no significant match was found. Species with no mRNA were queried with *T*. *truncatus* data.

### 2) Promoter Sanger sequencing

To confirm and further investigate the short duplications identified upstream from MHC class II genes in *T*. *truncatus* and *Orcinus orca* WGS data (*DQA*, *DRA*; see Results), primers were designed to amplify the proximal promoter regions of *DQA*, *DQB*, *DRA*, and *DRB* using Primer3 [80] and IDT OligoAnalyzer (http://www.idtdna.com/analyzer/applications/oligoanalyzer/). Primers are shown in Table 5 and PCR conditions are shown in S3 Table. Amplicons were confirmed using gel electrophoresis; 10 μl PCR product was purified with 1.5 μl shrimp alkaline phosphatase and 1.0 μl Exonuclease I in 20 μl reactions at 37°C for 30 min and 80°C for 15 min. Sequencing reactions were performed in 8.5 μl volumes using 3.5 μl of purified PCR product, 1.5 μl BigDye v3.1, and 3 μl of 1 μM forward or reverse primer (two reactions per sample). The sequencing profile was 96°C 1 min, 96°C 10s, 50°C 5s, and 60°C 4 min x35 cycles. A standard ethanol precipitation protocol was performed followed by suspension in 10 μl HIDI^TM^ formamide and a 2 min 90°C heat shock. Both 5’ and 3’ sequencing were performed on each sample using the ABI 3130xl Genetic Analyzer and v3.1 Dye Terminator Cycle Sequencing. Sequencing was performed on *T*. *truncatus* (North West Atlantic, n = 10) and *O*. *orca* (North West Pacific, n = 1), as well as *Delphinapterus leucas* (North East Pacific, n = 4).

**Table 5:** Primers and product sizes.

Description	Primer Pair	Forward 5'-3'	Reverse 5'-3'	Product Size (bp)
***DQA* Promoter**	F_3381 R_3640	GCCTCAGAACCAAGGGATTT	GAGGGTCCCCAGAATCAGAG	260
***DQB* Promoter**	F_5671 R_5967	TTCACCCGAAATGTTCATCC	AAGGCCTCTGGGGATCTG	297
***DRA* Promoter**	F_49 R_397	TCAGGGAGATCCATTTCTGG	GGTGTCTCGATGAGGGTCAG	349
***DRB* Promoter**	F_9828 R_10164	GCTCTCAAGAGAAGCCCAAA	CCGGAGAAATACAGGGACAC	337
***DQA* PBR**	F_7582 R_8110	GCCCGTCACCTTCACTTATC	GCTTGTTAAGGAGGGAGGTC	529
***DQA* Promoter NGS**	F_3286 R_3640	TCACCAGCAGGCATACACAT	GAGGGTCCCCAGAATCAGAG	352
***DQB* Promoter NGS**	F_620 R_977	ACCCGAAATGTTCATCCAGT	AGTCTCTGCCCTCAGCCTCT	358
***DQA* PBR NGS**	F_7582 R_7934	GCCCGTCACCTTCACTTATC	TCTCCTTAGGGAACAAGAGA	350
***DQB* PBR NGS**	F_2391 R_2726	GCTGAGCGGCGGTGTCT	CCCTGCGCGGAGTCTCG	352

NGS indicates the primers were used for Ion Torrent sequencing and thus also had 10 bp barcodes attached (Ion Xpress Barcode Adaptors verified list).

### 3) *DQA* PBR Sanger sequencing, cloning, and genotyping

The *DQA* PBR was isolated and sequenced in-house from a subset of *T*. *truncatus* samples (n = 88) using traditional Sanger sequencing to ground truth NGS methods described in the next section. Primers were designed in Primer3 and checked for hairpins and primer dimers using IDT OligoAnalyzer. The complete exon 2 was then captured using the primers F_7582 and R_8110 for a product size of 529 bp (Table 5). S2 Fig illustrates the start/end of the exon 2 coding region, which had not been fully captured previously. PCR conditions can be found in S3 Table. PCR products were prepared and sequenced as described above. Of 88 samples, 66 passed manual quality control (chromatograms had high signal/low noise, clean peaks; S3 Fig) for overlapping forward and reverse reads and were manually aligned using Sequence Analysis v5.2. Heterozygotes were identified from double peaks appearing in both forward and reverse strands of a sample. A representative subset of the heterozygous individuals (containing all potential variable sites, n = 10) were then cloned in order to type the alleles, alongside a homozygous individual for quality control. To ensure the highest confidence in the data, each sample was independently extracted two times and then each extraction was PCR amplified, cloned, and sequenced in replicates of three. Samples were PCR amplified as describe above in 50μL reactions and verified by gel electrophoresis. PCR products were purified with a MinElute PCR Purification Kit. A TOPO-TA Cloning® Kit was used with One Shot TOP 10 Chemically Competent *E*. *coli* and cells were plated with X-gal and ampicillin overnight. For each sample, 10 to 20 colonies were selected and grown overnight with LB broth and ampicillin. Culture was used as PCR template with M13 forward and reverse primers as described in S3 Table. Products were verified and purified, followed by a sequencing reaction with M13 primers and ethanol precipitation. Sequences were aligned against a homozygous individual used as the template in SeqScape v2.5 (Applied Biosystems) and seven alleles were typed and compared to the original Sanger sequences. The final cloned alleles were typed from at least two individual dolphins with the exception of Tutr-DQA1*07, which was only identified in one individual but was also observed in the NGS data described below. Typed alleles were imported to MEGA v5.0 [81] and aligned using ClustalW to verify that they were unique alleles. Alleles were converted to amino acid sequences to ensure that no stop codons were present, indicating that all sequences could form functional molecules.

### 4) DQ promoter and PBR NGS sequencing

A high throughput method was employed to collect more data which was verified by including the same samples from the standard traditional cloning-sequencing approach described above. The sample set was expanded to 95 dolphins from the four geographic strata in Fig 1: coastal Atlantic (ATL, n = 23), Mosquito Lagoon (ML, n = 24), North Indian River Lagoon (NIRL, n = 18), and South Indian River Lagoon (SIRL, n = 30). Four distinct PCR products were isolated, the proximal promoter region and the exon 2 PBR for both the *DQA* and *DQB* genes. Primer pairs (Table 5) were created with Primer3 and IDT OligoAnalyzer to amplify approximately 350 bp fragments across each region of interest for Ion Torrent™ sequencing and genotyping. All primers were tested with PCR, gel electrophoresis and Sanger sequencing to verify amplification of the desired targets prior to ordering barcoded sets of primers. A mixed barcodes approach was used such that 20 barcoded primers could cover 100 samples (10 barcoded forward primers x 10 barcoded reverse primers). Barcodes were selected from the Ion Torrent™ verified list (Ion Xpress Barcode Adaptors 1–96), using only ones which were exactly 10 bp (for ease of data processing) and which did not end in the same base as the first target sequence base (Ion Torrent recommendation). Five dolphins were selected as internal controls; each was run twice with different barcode combinations such that they underwent the whole process from PCR to data analysis separately and thus could be used to verify protocol consistency. PCR reactions were performed as described in S4 Table for each primer pair using phusion High-Fidelity DNA polymerase prepared in 20 or 40 μl reactions. PCR product size was verified using gel electrophoresis, followed by purification using Agencourt AMPure XP and elution into water. Samples were quantified using the dsDNA HS Assay with the Qubit 2.0, and 0.25 ng of each sample was combined for sequencing. The pooled samples were sent to University of Florida Interdisciplinary Center for Biotechnology Research (http://www.biotech.ufl.edu/) for library preparation using the Ion PGM Sequencing 400 Kit and Ion OneTouch 2 System, then loaded onto a Ion 318 Chip for sequencing on an Ion PGM System.

Raw fastq data were first parsed by length using USEARCH v1.0.1090 [82] to remove truncated reads unlikely to carry barcodes at each end. Expected length was amplicon length (~350 bp) plus two ten bp barcodes, but all reads with a minimum of 350 bp were kept to allow for potential deletions. FASTX Barcode Splitter (FASTX-toolkit http://hannonlab.cshl.edu/fastx_toolkit/links.html) was used to segregate reads by their 5’ barcode with 0 mismatches into separate files. Each file was then segregated according to the 3’ barcode (0 mismatches). Because sequence library preparation involved ligating adapters onto either end of the barcoded amplicon, reads could be in either orientation. Reverse read files were edited using FASTX Reverse Complement with -Q33 flag (to specify the correct PHRED quality scores), then combined with the appropriate forward read sequence file according to barcodes. Lastly, each file was processed with FASTX Trimmer to remove the first and last ten base pairs (barcodes).

The steps described here (S5 Table) were performed for each *DQA*/*DQB* promoter/PBR target (four total) against the reference sequence from which the primers were designed. Each reference fasta sequence was prepared by creating a TMAP index (http://github.com/iontorrent/tmap), a SAMtools index (Li et al. 2009), and a Picard sequence dictionary (http://picard.sourceforge.net/). Reads were aligned to the appropriate reference using TMAP4 (version 3.4.1) to generate sequence alignment map (sam) files. Two different variant analyses were run from this point for quality control. The first relied on SAMtools (version 0.1.18) and BCFtools (version 0.2.0-rc7-47-g02a1fb3) to convert sam files to bam, sort and index bam files, generate mpileups (-B to exclude BAQ), and output variant call format (vcf) files. The second analysis began with Picard (version 1.87) to sort and convert sam to bam and index bam files. Unified Genotyper, part of the Genome Analysis ToolKit (GATK, version 2.8-1-g932cd3a) [83], was used for the variant analysis with no downsampling. Variable sites from both analyses (SAMtools and UnifiedGenotyper) were phased using GATK ReadBackedPhasing to obtain two alleles for heterozygous samples (-dt NONE), and BCFtools was used to maneuver variant call format into a more readable format. Finally, VCFtools was used to convert phased vcf files into two fasta sequences (two alleles) per sample. BioEdit and Tablet (version 1.13.12.17) were used to visualize fasta and bam/sam files [75, 84]. The programs used for all analyses are freely available and shown in S6 Table. All newly typed alleles were named using the proposed method from Klein et al. [53].

### 5) Population and cross-taxa analyses

Neighbor Joining Trees were created using MEGA6 to depict similarity between alleles. Prior to examining the coding regions, the alleles were trimmed down to remove incomplete codons. Both *DQA* and *DQB* have disconnected codons (i.e., a codon begins on exon 1 and ends on exon 2) such that the first two and last base were removed from the alleles prior to analyses. *DQA* exon 2 is 249 bp but has only 82 complete aa, and *DQB* exon 2 is 270 bp but has only 89 complete aa. Following the antigen binding pockets identified for humans [58], 15 aa determine the conformation of the 3 DQA pockets P1, P6, and P9. Within *DQB*, 19 aa form the 5 pockets P1, P4, P6, P7, and P9 [13, 58]. Chi-Square and G-tests were performed to test independence of amino acid variation within antigen vs non-antigen binding sites. MHC variation is expected to be higher at the sites that directly interact with pathogenic peptides, thus these tests will examine if amino acid variation occurs equally at the aa sites forming the binding pockets and those outside the pockets. A maximum likelihood estimate of transition/transversion bias [85] was calculated across the coding regions using MEGA6 [86]. The d_N_:d_S_ ratios were calculated with a maximum likelihood model in CodeML [87] to test for evidence of positive selection. This ML estimator is better at accounting for potential biases in codon usage and transition/transversion rates than approximate estimators. Multiple phylogenetic trees of distinct DQ alleles were tested, both user tree and pairwise runmodes were employed, and the discrete site option for NS sites and model one option for codons were used. The Bayes empirical Bayes test in PAML v4.6 [87] determined codon sites likely under positive selection across the entire exon 2 of DQA and DQB genes, which were compared to binding pocket sites.

Population differentiation was examined using Arlequin v3.5 [88]. The frequency-based statistic, F_st_ [89], was calculated using the method of Weir and Cockerham [90] among the following geographic groupings: 1) Atlantic, 2) Mosquito Lagoon, 3) North IRL, 4) South IRL, 5) IRL (NIRL and SIRL), and 6) Estuarine (NIRL, SIRL, and ML) (Fig 1). Homogeneity tests were conducted via 10,100 permutations of the data. Deviations from Hardy-Weinberg equilibrium (HWE) were tested using the exact test with p-values estimated from 10,000 iterations of the data using the Markov chain method [91]. DnaSP v5.10.01 [92] was used to evaluate the average Codon Usage Bias Index (CBI), the effective number of codons (ENC), the relative synonymous codon usage (RSCU) and GC content of *DQA* and *DQB* PBR. Finally, promoter polymorphism was also examined within dolphins and across cetaceans using Shannon entropy [15]. Because these regions are non-coding, there are few analyses available for evaluating the evolutionary forces at play or for characterizing the importance of conservation versus diversification. Cowell et al. [15] suggest that entropy measurements may serve as an effective indicator through highlighting sites of conservation at higher taxa levels.

“This article does not contain any studies with human participants or animals performed by any of the authors.”

## Supporting information

S1 Fig Promoter/Transcription factor/CIITA complex. Highly conserved transcription factor binding motifs (S, X1/X2, and Y) are in the proximal promoter regions of all MHC class II genes. These motifs must be bound by transcription factors, which in turn are bound by the class II transactivator (CIITA) before transcription can begin.
(PNG)

S2 Fig Primers for amplifying DQ promoter regions and peptide binding region. Primers developed and used in this study are indicated by capital letters, lower case reference other studies as shown in legend. **A)** Distances are based on *Tursiops truncatus* WGS gi|366536137 and GENSCAN exon/intron boundaries for DQA. **B)** Distances are based on *T*. *truncatus* WGS gi|366536136 and mRNA Tutr-DQB EF017815.
(PNG)

S3 Fig Aligned chromatograms of three sanger sequenced individuals. Boxes indicate variable sites and homozygous/heterozygous calls are noted at these sites.
(PNG)

S4 Fig NGS sequencing error in DQA peptide binding region. Sites 206, 215, and 222 were called by SAMTools and confirmed by Sanger sequencing. Sites 216 and 228 (boxed) were only called variable by UnifiedGenotyper but were not confirmed with Sanger, nor were these sites variable in any other sample, thus they likely represent sequencing error.
(PNG)

S5 Fig NGS Chimera Error in DQA peptide binding region. Sites 172 and 175 occur on one allele, while sites 206, 215, and 222 occur on another. PCR chimera formation resulted in a unique allele artefact (box). SAMTools missed several variable sites due to sensitivity to allelic ratios, which was thrown off by the false allele. UnifiedGenotyper correctly called the alleles which were confirmed by Sanger sequencing.
(PNG)

S6 Fig NGS alignment error in DQB peptide binding region. Deletions (blue box) occurred in the Forward sequencing reaction at site 176 for one allele. TMAP incorrectly called another deletion (site 177) and an insertion (red box, between sites 181 and 182). Miscalls were made from both SAMTools (178, 179, 180, 181) and UnifiedGenotyper (180). Correct calls can be made from the data by excluding forward reads.
(PNG)

S7 Fig NGS Triallelic Error in DQB peptide binding region. Site 88 (box) was either C or T in all samples, but SAMTools always calls one allele as the reference (G). UnifiedGenotyper correctly called both variations, however the phase had to be manually corrected.
(PNG)

S8 Fig NGS homopolymer error in DQB promoter region. Site 178 (box) was T in nearly all reads, yet UnifiedGenotyper could not call a variant inside of a homopolymer (changing T_4_AT_2_ to T_7_). SAMTools correctly called site 178 as verified by Sanger sequencing.
(PNG)

S9 Fig *DQA* promoter region alleles. Six alleles were typed for *Tursiops truncatus DQA* proximal promoter region. Transcription factor binding motifs are indicated at the top of the alignment as needed, including the duplicated T-box (upstream to the original T-box). GenBank #MG211347-MG211352.
(PNG)

S10 Fig *DQB* promoter region alleles. Seven alleles were typed for *Tursiops truncatus DQB* proximal promoter region. Transcription factor binding motifs are indicated at the top of the alignment as needed. GenBank #MG211363-MG211369.
(PNG)

S11 Fig *DQA* peptide binding region alleles. Ten alleles were typed for *Tursiops truncatus DQA* exon 2. For reference, the exon 2 sequence from the WGS is shown at the bottom. GenBank #MG211337-MG211346.
(PNG)

S12 Fig *DQB* peptide binding region alleles. Ten alleles were typed for *Tursiops truncatus DQB* exon 2. For reference, the exon 2 sequence from the WGS is shown at the bottom. GenBank #MG211353-MG211362.
(PNG)

S13 Fig Neighbor joining trees. Unrooted trees were computed for each target region to illustrate similarity between alleles in *Tursiops truncatus*.
(PNG)

S1 Table Summary of Tutr-DQB alleles. All Tutr-DQB alleles (n = 51) were downloaded from GenBank, aligned with the 10 alleles described here, and trimmed to the shortest shared region (169 bp) to determine similarity. The source country is shown if available; parentheses indicate that the sample country was not provided, but rather show the location of the research institution.
(XLSX)

S2 Table Summary of Tutr-DQA alleles. All Tutr-DQB alleles (n = 17) were downloaded from GenBank, aligned with the 10 alleles described here, and trimmed to the shortest shared region (227 bp) to determine similarity. The source country is shown for each sample.
(XLSX)

S3 Table PCR reaction conditions for Sanger sequencing. Conditions were uniform across the first four primer pairs from Table 5 and used for Sanger Sequencing in dolphin, orca, and beluga (Section 2 in Methods). The DQA Peptide Binding Region (PBR) was PCR amplified in dolphin, and a subset was cloned. Colonies were used for M13 PCR and sequencing (Section 3 in Methods). Refer to Table 5 for primers.
(XLSX)

S4 Table PCR reaction conditions for next generation sequencing. Four regions of interest were amplified in dolphins with barcoded primers. Amplicon were purified, pooled, and sent for Ion Torrent sequencing (Section 4 in Methods). Refer to Table 5 for primers.
(XLSX)

S5 Table Data analysis pipeline. Each step is shown with the relevant program and command options used. Two separate variant analyses were performed on the prepared reads (SAMtools and UnifiedGenotyper).
(XLSX)

S6 Table Programs for data analyses. All programs are freely available for download.
(XLSX)
