# Supplementary figures and images for: Positive selection in coding regions and motif duplication in regulatory regions of bottlenose dolphin MHC class II genes
Source: PLoS One. 2018 Sep 25;13(9):e0203450. doi: 10.1371/journal.pone.0203450 (PMC6155461; doi:10.1371/journal.pone.0203450)

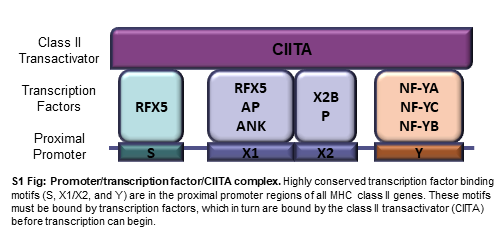

Supplement: S1 Fig — Highly conserved transcription factor binding motifs (S, X1/X2, and Y) are in the proximal promoter regions of all MHC class II genes. These motifs must be bound by transcription factors, which in turn are bound by the class II transactivator (CIITA) before transcription can begin. (PNG) [file pone.0203450.s001.png]

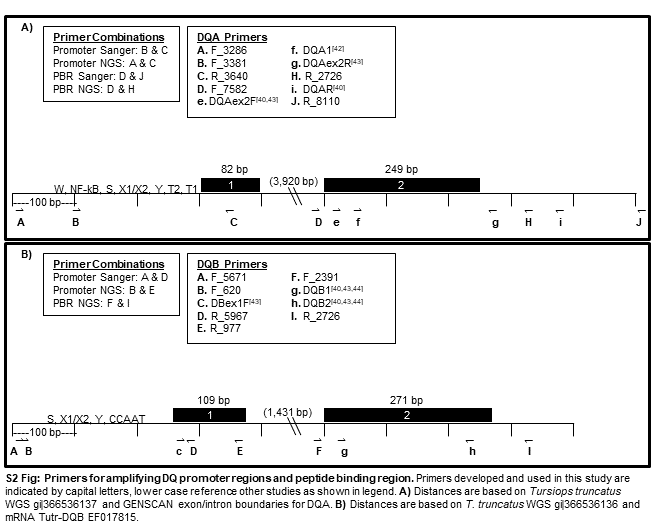

Supplement: S2 Fig — Primers developed and used in this study are indicated by capital letters, lower case reference other studies as shown in legend. A) Distances are based on Tursiops truncatus WGS gi|366536137 and GENSCAN exon/intron boundaries for DQA. B) Distances are based on T. truncatus WGS gi|366536136 and mRNA Tutr-DQB EF017815. (PNG) [file pone.0203450.s002.png]

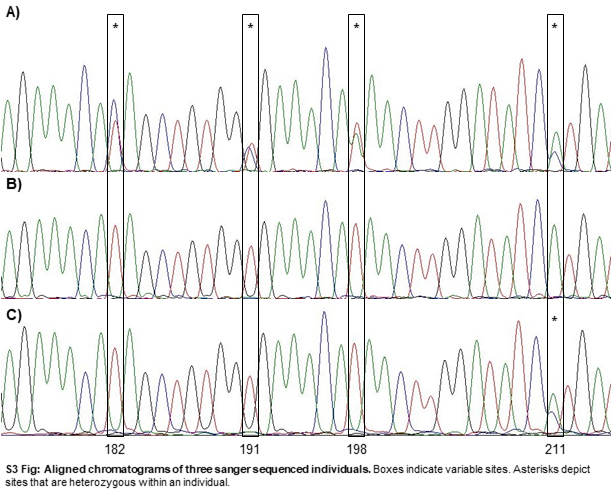

Supplement: S3 Fig — Boxes indicate variable sites and homozygous/heterozygous calls are noted at these sites. (PNG) [file pone.0203450.s003.png]

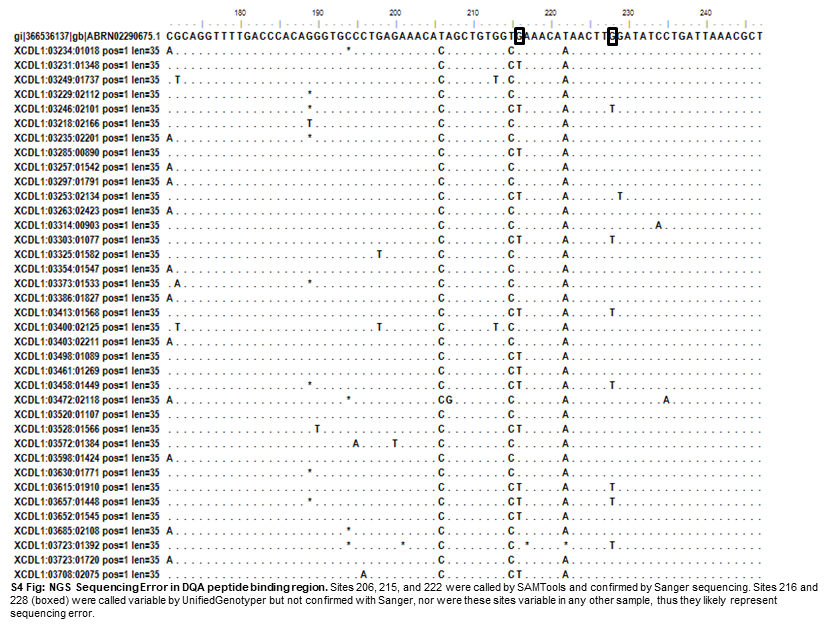

Supplement: S4 Fig — Sites 206, 215, and 222 were called by SAMTools and confirmed by Sanger sequencing. Sites 216 and 228 (boxed) were only called variable by UnifiedGenotyper but were not confirmed with Sanger, nor were these sites variable in any other sample, thus they likely represent sequencing error. (PNG) [file pone.0203450.s004.png]

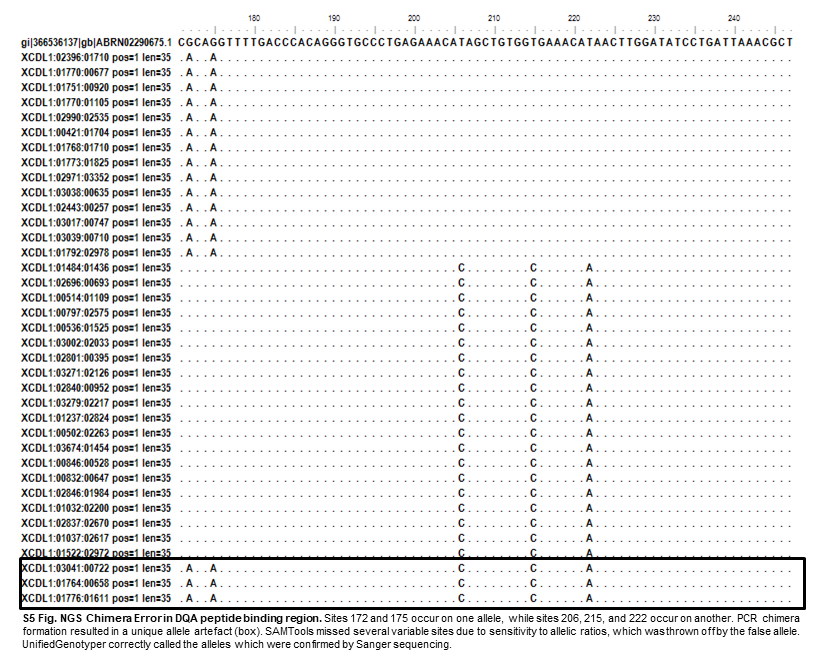

Supplement: S5 Fig — Sites 172 and 175 occur on one allele, while sites 206, 215, and 222 occur on another. PCR chimera formation resulted in a unique allele artefact (box). SAMTools missed several variable sites due to sensitivity to allelic ratios, which was thrown off by the false allele. UnifiedGenotyper correctly called the alleles which were confirmed by Sanger sequencing. (PNG) [file pone.0203450.s005.png]

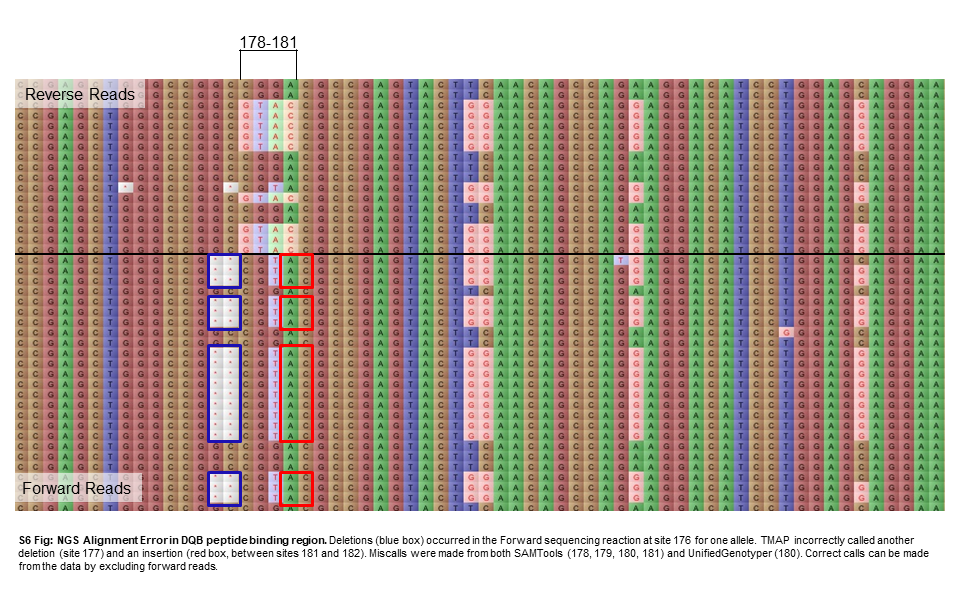

Supplement: S6 Fig — Deletions (blue box) occurred in the Forward sequencing reaction at site 176 for one allele. TMAP incorrectly called another deletion (site 177) and an insertion (red box, between sites 181 and 182). Miscalls were made from both SAMTools (178, 179, 180, 181) and UnifiedGenotyper (180). Correct calls can be made from the data by excluding forward reads. (PNG) [file pone.0203450.s006.png]

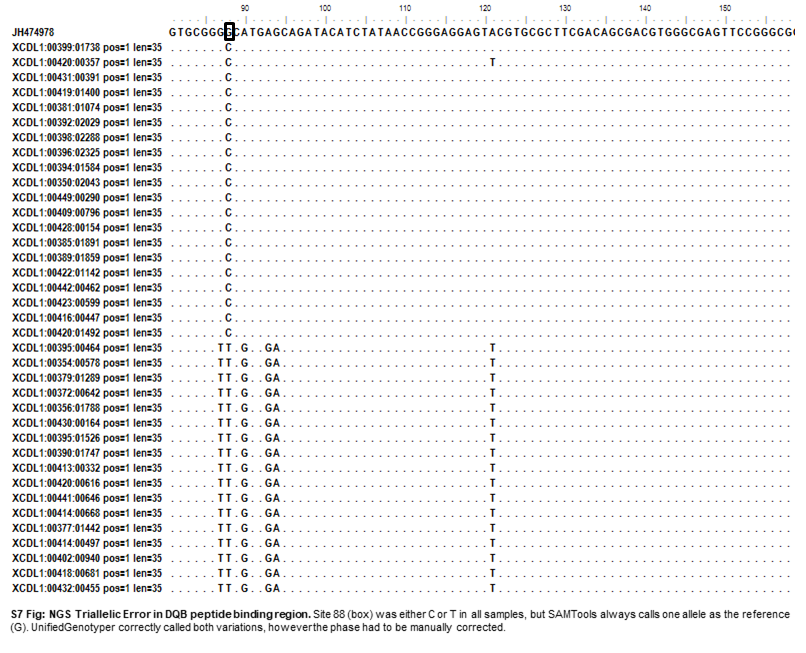

Supplement: S7 Fig — Site 88 (box) was either C or T in all samples, but SAMTools always calls one allele as the reference (G). UnifiedGenotyper correctly called both variations, however the phase had to be manually corrected. (PNG) [file pone.0203450.s007.png]

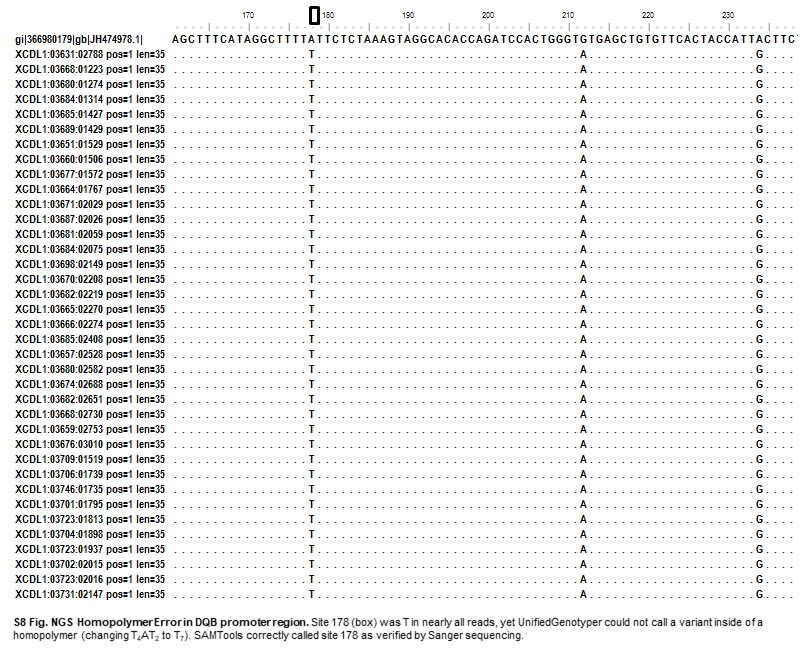

Supplement: S8 Fig — Site 178 (box) was T in nearly all reads, yet UnifiedGenotyper could not call a variant inside of a homopolymer (changing T4AT2 to T7). SAMTools correctly called site 178 as verified by Sanger sequencing. (PNG) [file pone.0203450.s008.png]

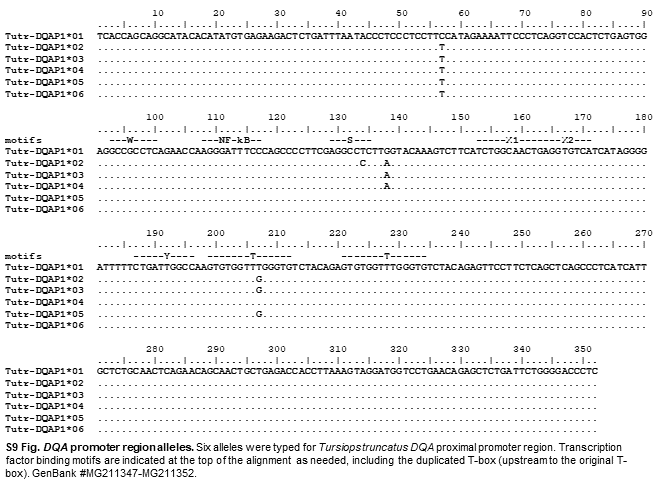

Supplement: S9 Fig — Six alleles were typed for Tursiops truncatus DQA proximal promoter region. Transcription factor binding motifs are indicated at the top of the alignment as needed, including the duplicated T-box (upstream to the original T-box). GenBank #MG211347-MG211352. (PNG) [file pone.0203450.s009.png]

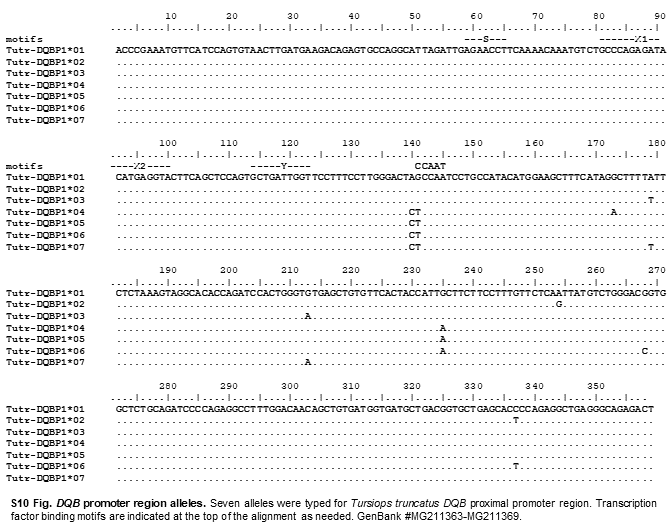

Supplement: S10 Fig — Seven alleles were typed for Tursiops truncatus DQB proximal promoter region. Transcription factor binding motifs are indicated at the top of the alignment as needed. GenBank #MG211363-MG211369. (PNG) [file pone.0203450.s010.png]

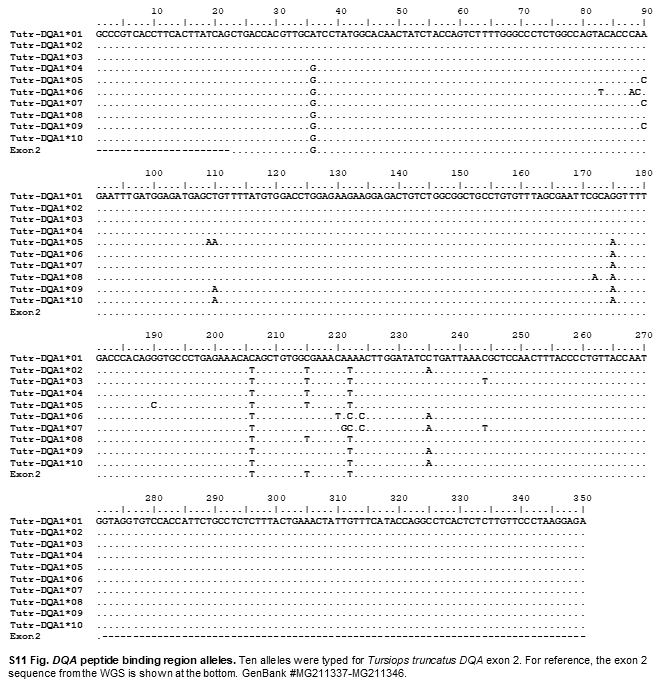

Supplement: S11 Fig — Ten alleles were typed for Tursiops truncatus DQA exon 2. For reference, the exon 2 sequence from the WGS is shown at the bottom. GenBank #MG211337-MG211346. (PNG) [file pone.0203450.s011.png]

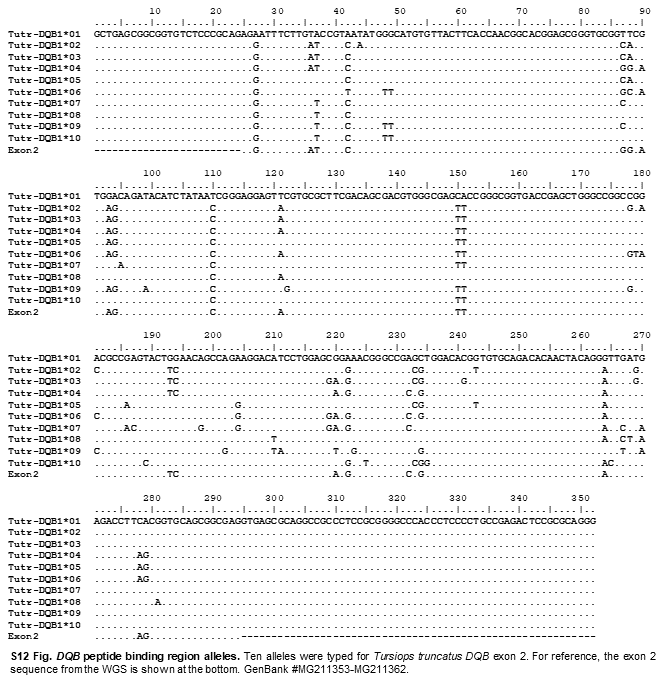

Supplement: S12 Fig — Ten alleles were typed for Tursiops truncatus DQB exon 2. For reference, the exon 2 sequence from the WGS is shown at the bottom. GenBank #MG211353-MG211362. (PNG) [file pone.0203450.s012.png]

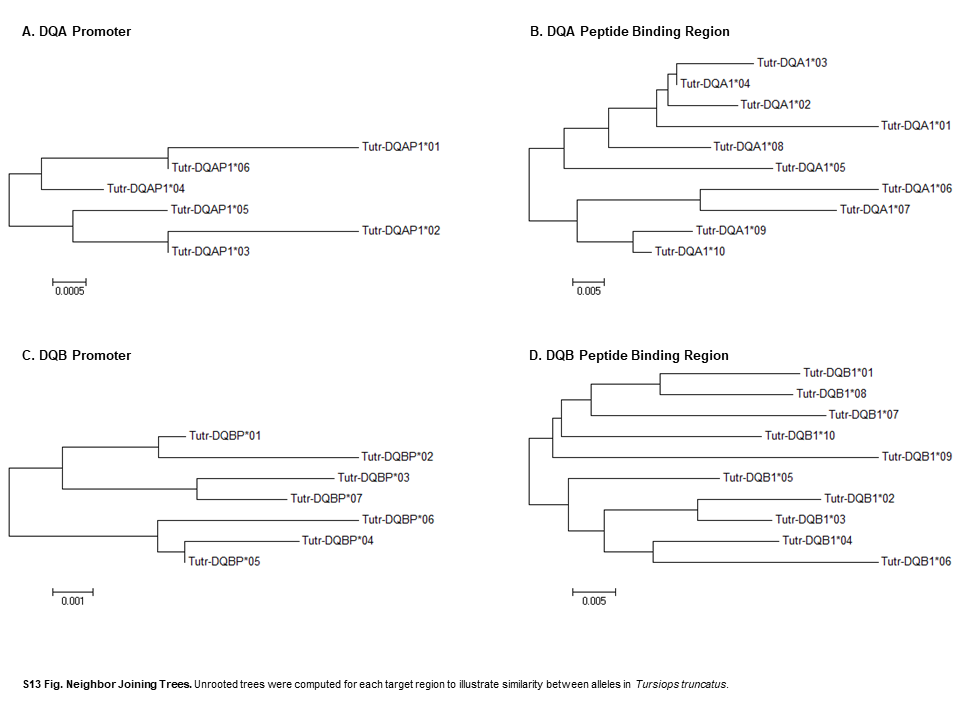

Supplement: S13 Fig — Unrooted trees were computed for each target region to illustrate similarity between alleles in Tursiops truncatus. (PNG) [file pone.0203450.s013.png]
